# A Temple of Lucifer

**Published:** 1888-04-07

**Authors:** 


### A Temple of Lucifer.

"Dear reader, can you—can you remember the days of the tinder-box?" asks Wendell Holmes, in one of his delightful talks on things in general. The number of persons who could answer in the affirmative must now be very small. The lucifer match has become one of those daily comforts which are so common that we are all, even the poorest of us, grandly ungrateful for them. The lucifer match! The first part of the name is dropped now; the supposed diabolism of the flame that springs from the friction of brimstone with sand-paper is forgotten by this generation, and a novelist is accused of an unnecessary concession to realism when he makes his hero allude, in a romance of South Africa, to obtaining a light by striking one of Bryant and May's matches.

I wonder if Mr. Rider Haggard took the trouble, among his other studies for the production of "She," to learn how these matches of Bryant and May's are made. Probably not; yet the large manufactory at Bow is well worth a visit. It consists of several large and solidly-constructed buildings standing in about ten acres of ground; but in spite of the accommodation thus afforded, there are two details of the manufacture which are not carried on here. The wood is cut up into the "splints" for making the matches at supplementary works at Bow Common, and the making of boxes goes on in various quarters. The company which now bears the name of Bryant and May, Limited, find that they can import the boxes from the Continent cheaper than they can have them made in this country; but they still provide a good deal of work of this kind for the women of Bethnal Green. The price they give for this has been the subject of much animadversion. It is from 2¼*d.* to 2¾*d.* a gross. This certainly seems a small sum for twelve dozen of match-boxes; but they can be made very quickly, and one of the directors of the company informed me that women who gave all their working time to the making of these boxes could earn from twelve to fifteen shillings a-week. Box-making can be carried on at home, and women who could not leave their children to go out to work can thus earn a few shillings a week and still attend to their households.

These boxes are stored on the upper floors of the factory, but before we reach these let us have a look at the processes by which our matches are produced. In the first place, the worker having supplied herself with splints, each the length of two matches, feeds with them a machine which dexterously winds them up with a strip of belting into a wide circular cake, the belting going between the rows to keep the splints at a sufficient distance from each other to prevent them adhering to each other when they are dipped in the fire-producing mixture. This cake being securely tied up, the ends of the splints are placed for a moment in a mixture of paraffin, which saturates the wood to some extent. When one end is thus moistened, the cake is placed on a hot iron plate to dry, and then the other side is treated in the same way. When this is done the splints are dipped in the various mixtures that form the head of the match—the dark-brown of the well-known safety matches, or the red or blue of the cheaper kinds, and are then placed in the drying-rooms which line each side of the large room where the dipping process goes on. These drying-rooms are small, with thick walls, and closely-fitting iron doors; so that in the event of any fire arising it could be confined to the room where it originated. It is needless to say, however, that the heat in these rooms is very moderate; and there is always plenty of water at hand; in fact, in any part of the factory where inflammatory substances are in use the floors are kept continually wet.

The matches being thus prepared, but each tipped at both ends, and double the ordinary length, they are unwound from the belting and handed to the girls who arrange them in the boxes. These take a handful—and it is amazing how accurately they guess just the number it requires to fill a box—place them beneath a sort of guillotine, whose knife cuts them in half, and in an instant two boxes are filled. The swiftness with which the girls work explains how they can earn good wages by work which, when the price by the piece is mentioned, seems very ill paid.

The wax vestas and braided lights are tipped in the same manner, but with these some of the preparatory stages of manufacture are carried on at Bow. The splints for the braided lights are prepared at the works, not in single lengths, but in very long strips, so thin that the wood is tolerably flexible. One of these strips is pushed up a hole in the centre of an iron plate just between two wires; while on the outside about half-a-dozen bobbins of yellow cotton are "setting to partners" and dancing in and out till the threads are twisted a somewhat similar fashion to the may-pole dances of the stage, binding the wires close to the wooden splint. It is the presence of these wires that makes the head of the braided light adhere better than that of the ordinary vesuvian, and thus win the approval of smokers.

The wax vestas are made on the premises *ab initio*, and their manufacture is a very interesting process. The stearine—what the outside world calls wax, using the name of one of its constituents—having been purified and melted, is divided among several baths, and the threads of cotton which form the foundation of the taper are drawn through these. Formerly only one thread at a time was so surrounded by the stearine, but now, by an ingenious machine, the strands of cotton yarn, in a bale weighing two-and-a-quarter cwt., are divided into a hundred threads of equal thickness and pass through a plate, the holes in which are of the exact size the taper is to be. The hundred strands of cotton having thus received one bath are dried and wound by machinery on a large drum capable of holding many miles of taper. They pass through another bath and then another till they attain the required diameter, and after that are cut by an ingenious machine into the proper lengths, fixed in a frame, and dipped in the usual way. With the vestas, unlike the wooden matches, the full frame is given to the packer, who loosens the rows as she wants them, as, being of a different material, they will not stand such rough usage.

As we were passing through the room where most of the boxing of the vestas was done, the foreman said, "You must come and see my nursery," and took us into a side room where about twenty young girls not long from school were at work. They were slower and clumsier than their seniors, "But," said the foreman, "if I let them go into the big room at once the elder girls won't show them all the details of the work, but use them to run errands." So he takes them under his special care, and sees that they are treated with due patience until they get used to the work. Very happy and cheerful they seemed over it; and indeed all the workers in the factory seemed well enough contented with their lot, and certainly they were exceptionally rosy and healthy-looking for London girls, who are mostly accustomed to work-rooms less fresh and well ventilated than Messrs. Bryant and May's.

Besides the making of matches, both for home and foreign consumption, the firm has a patent for the printing of tin. Beginning with making fancy boxes of various kinds for their matches and vestas, the business has extended in various directions, and now their trays, tea-canisters and candlesticks are to be seen all over the country, and those who buy them never guess that they are supplied by the great match firm. The designs of many of these are of considerable artistic merit; all show taste and good workmanship, wonderful to be obtained at so low a price. Throughout the factory one is struck with the steady, conscientious industry that goes on. How is this secured? By exceptionally vigilant supervision? No; by something more efficient—co-operation. Most of the foremen are shareholders in the company, and therefore, as is natural to mortals, see to everything carefully. The success of the firm of "Bryant and May, Limited," may suggest the solution of some troublesome trade problems, and it is probable that the more thoroughly they carry out the principle of making the leading workmen sharers in the profit of the work, the greater their success would be.

